# Awareness and Vaccine Coverage of Hepatitis B among Cameroonian Medical Students

**DOI:** 10.1155/2018/3673289

**Published:** 2018-09-25

**Authors:** Desmond Aroke, Benjamin Momo Kadia, Ephesians Nkwetta Anutebeh, Cluade Asaba Belanquale, Glory Masango Misori, Alfred Awa, Clarence M. Mbanga, Larry Tangie Ngek

**Affiliations:** ^1^Fontem District Hospital, Fontem, Cameroon; ^2^Health and Human Development (2HD) Research Network, Douala, Cameroon; ^3^Grace Community Health and Development Association (GRACHADA), Kumba, Cameroon; ^4^Faculty of Infectious and Tropical Diseases, London School of Hygiene and Tropical Medicine, London, UK; ^5^Faculty of Health Sciences, University of Buea, Buea, Cameroon; ^6^Faculty of Medicine and Biomedical Sciences, University of Yaoundé I, Yaoundé, Cameroon; ^7^Ndop District Hospital, Ndop, Cameroon; ^8^Mankon Sub-Divisional Hospital, Bamenda, Cameroon

## Abstract

**Background:**

The endemic nature of the hepatitis B virus (HBV) in Sub-Saharan Africa is a significant public health problem that places health care providers (medical students inclusive) at increased risk of occupational exposure. However vaccination against HBV is not systematic among medical students in Cameroon. Thus, we sought to evaluate awareness and HBV vaccine coverage among medical students in Cameroon.

**Methods:**

Using semistructured questionnaire and a cross-sectional approach, medical students from 3 State Universities in Cameroon were evaluated for their knowledge, attitudes, and vaccination status against the HBV. Data were collected over a 3-month period. HBV vaccine status was defined as complete (3 doses), partial (1 and 2 doses), and unvaccinated. Data were entered and analyzed using Epi-info 7.

**Results:**

There were 714 respondents among whom 186 (26.05%) had been vaccinated at least once against HBV. Sixty-six (9.24%) were partially vaccinated and 120 (16.81%) completely vaccinated. No student had done postvaccination serologic testing to confirm full immunisation. Eighty-three percent (83.00%) of respondents had adequate knowledge on HBV infection and vaccine, while 90.00% had adequate knowledge on HBV transmission. Most medical students had a positive attitude towards the HBV vaccine. The most common negative attitudes were worries about the side effects and fears of being infected by the vaccine.

**Conclusion:**

Despite adequate knowledge on HBV infection and vaccination only about 1 in 6 medical students had completed the HBV vaccination series. This highlights the need for better health policies aimed at increasing access and coverage of the HBV in at-risk populations like medical students.

## 1. Background

Hepatitis B virus (HBV) is a DNA virus of the family* hepadnaviridae* which could be responsible for acute and/or chronic pathology in the liver. Globally, two billion people (about one-third of the world's population) have serological evidence of prior HBV infection. An estimated 240 million people (4% of the world's population) are chronic HBV carriers, with about 780,000 deaths occurring yearly from HBV related causes [1]. In Sub-Saharan Africa, HBV is highly endemic with a prevalence of 5 – 10%. In Cameroon, HBV remains an important public health problem with a prevalence rate of about 8% [2].

HBV can be transmitted through percutaneous or mucosal exposure to infected blood and bodily fluids. Health care providers (HCP) and/or medical students have been shown to be four times more likely to contract the HBV than the general adult population [3].This is because the health care environment and the nature of their work increases their risk of being exposed to infection with blood-borne pathogens such as HBV, hepatitis C virus (HCV), and human immunodeficiency virus (HIV) [4, 5]. HBV is highly infectious and is 40 to 100 times more contagious when compared to HIV [6]. HBV can remain stable on environmental surfaces for up to 7 days. It is thus the greatest threat of infection for HCP [7].

HBV infection can be prevented by following a simple and available vaccination schedule [8]. HBV vaccine is the first anticancer vaccine which has outstanding record of safety and effectiveness. It is 95% effective in preventing children and adults from developing chronic infection [1]. More so, routine vaccination of HCPs in the United States of America (USA) has been demonstrated to decrease the incidence of HBV [9–11]. Despite the high prevalence of HBV and effectiveness of the vaccine, there is no policy on routine vaccination of HCPs in Cameroon. The Cameroon government in a bit to reduce the high prevalence of HBV since 2004 systematically provides HBV vaccine to all infants as an integral part of the Expanded Programme of Immunisation at 6 weeks, 10 weeks, and 14 weeks [12]. However, this measure fails to protect the exposed adult population. Awareness of transmission routes, risk involved in treatment procedures, and implementation of appropriate precautions when treating patients are critical in preventing the spread of this infection. We therefore set to evaluate the knowledge of HBV, attitudes towards HBV vaccine, and vaccine coverage of HBV vaccine among medical students.

## 2. Methods

### 2.1. Study Design, Setting, and Participants

This was a descriptive cross-sectional survey carried out over a period of 3 months (August 2016 – November 2016) among students in Faculty of Health Sciences of the Universities of Buea and Bamenda and the Faculty of Medicine and Biomedical Sciences of the University of Yaoundé I. Medical schools were randomly chosen while recruitment of participants in each medical school was by convenient sampling. A medical student from each medical school was contacted by the principal investigator and trained on data collection procedure. In each medical school, the data collector visited each classroom during break, approached participants, and proposed the study. Participants who were present on days of the study and who gave written consent to participate in the study were recruited. Participants were approached and explained the nature and purpose of the study. Participants with a known positive hepatitis B status were excluded from the study. Description of this cross-sectional survey was based on the STROBE statement [13].

### 2.2. Ethical Considerations

The study received ethical approval from the Institutional Review Board of the Faculty of Health Sciences of the University of Buea. Consent forms were signed by students who agreed to participate in the study. Confidentiality was ensured during the study.

### 2.3. Data Collection

A pretested semistructured pen and paper questionnaire was given to each participant to fill. Data were collected on sociodemographic characteristics, knowledge on routes of transmission and signs and symptoms of HBV infection, attitudes, and practices towards hepatitis B vaccine.

### 2.4. Definition of Operational Terms and Variables

Scores of ≥ 8/12 and ≥ 5/7 on correct knowledge defined adequate knowledge on HBV infection and vaccine, and HBV transmission, respectively. A positive attitude was based on a score of ≥ 4 of 7 positive attitudes while a negative attitude was based on a score of ≥ 3 of 6 negative attitudes. HBV vaccine status was defined as complete (3 doses), partial (1 and 2 doses), and not vaccinated (zero dose). See Table 1.

#### 2.4.1. Statistical Analysis

Data were entered and analyzed using Epi-info version 7 statistical software. Results are presented as counts (percentages), mean and standard deviation (SD), or median and interquartile range (IQR) as appropriate.

## 3. Results

### 3.1. Sociodemographic Characteristics

During the study period 758 medical students were approached while 714 medical students accepted to participate in the study. The retention rate was 94.20%. The mean age (SD) of participants was 22.03 (±1.1) years. Other characteristics are summarised in Table 2.

### 3.2. Rate of HBV Vaccine Uptake

Of 714 participants, 186 (26.05%) had been vaccinated at least once against HBV. Of these, 28 (3.92%) had been administered only the 1^st^ dose, 38 (5.32%) had received 2 doses, and 120 (16.81%) had received all 3 doses. Of the 186 students vaccinated at least once 102 (54.84%) were clinical year students and of the 120 who had completed vaccination 86 (71.67%) were in the clinical years. The complete vaccine uptake rate among clinical year students was 19.20% and among basic sciences students was 12.70%. Sixty-two (15.90%), 26 (15.29%), and 32 (20.78%) students of the medical schools of the universities of Buea and Bamenda and Yaoundé I, respectively, had been completely vaccinated.

### 3.3. Knowledge on HBV and Vaccine Uptake

Correct scores ranged from 3 to 12 with a mode of 9 correct scores. 83% of our participants had adequate knowledge on HBV infection and vaccine. 93% of participants correctly responded that health care providers can spread the infection to patients and 89% accepted that patients can spread the infection to them. Correct scores on HBV transmission ranged from 0 to 7 with a mode of 6 correct scores. 90% of our participants had adequate knowledge on HBV transmission. Also, 92% of respondents correctly responded that transmission was possible through broken skin in contact with contaminated blood.  Table 3 summarises responses to questions asked on HBV infection and vaccine.

### 3.4. Attitudes towards HBV and Vaccine Uptake

As shown on Table 4, most medical students had a positive attitude towards the HBV vaccine. The vaccine was available in a third of their work places. The main negative attitudes towards the vaccine were worries about the adverse effect (48.99%) and being afraid of infection from the vaccine (39.31%).

## 4. Discussion

Medical students are well known to be at increased risk of contracting HBV [14–16]. This study is one of the few studies in Cameroon that assessed the knowledge and vaccine coverage of medical students in state medical schools. It appears that medical students had a good knowledge on HBV infection, routes of transmission, and vaccine. Despite this awareness only 1 in 6 medical students had received the complete HBV vaccine and none of those vaccinated had done the postvaccination antibody titres to confirm immunisation. The vaccination uptake was understandably higher among clinical year students. The low rate of vaccine uptake was due to unavailability of the vaccine and possibly lack of policies.

Only one-quarter of the respondents had ever taken an HBV vaccine, and among all respondents, only about 1 in 6 had received the complete vaccine. Although most respondents had an appropriate knowledge on HBV infection, routes of transmission, and the vaccine, the vaccine coverage was low. Unavailability of the vaccine in most hospitals and lack of a clear policy on HBV vaccination in medical schools could in part explain the low vaccine coverage. Also some respondents had negative attitudes towards the vaccine which included worrying about side effects and risk of infection. HBV is a major public health concern and being one of the most contagious blood-borne infections should be given utmost attention. The 16.8% complete vaccination rate among our respondents is alarming given their increased risk of contracting HBV. Though similar vaccine uptake rates have been reported in India and Cameroon, these studies were done among first-year students and using smaller sample sizes [17, 18]. Much higher rates have been obtained in Nigeria, Bangladesh, Pakistan, and Brazil where there are more organised and structured programs to encourage uptake of the vaccine [16, 19–21]. Urgent measures are therefore required to put in place policies that will encourage vaccination coverage among students.

The Advisory Committee on Immunisation Practices (ACIP) has since 1997 recommended postvaccination serologic testing at least 1 – 2 months following last dose of vaccine to confirm immunity [15]. Previous studies among medical students have reported postvaccination serologic testing rates of 10 – 65% [18, 21, 22]. It is intriguing that none of our participants had measured antibody titres after vaccination. This is possibly because most people are unaware of the existence of the test and unavailability of the test in most tertiary centres of health care.

It is of paramount importance for medical students to be properly informed on HBV so as to protect themselves and their potential patients. Also they are in frequent contact with the general population and are thus expected to know and promote measures to immunise the general public. Generally, most (83%) medical students had a good knowledge on HBV infection and vaccination. However on a closer look it was disturbing to find out that up to 156 (22%) medical students did not know HBV is contagious. Majority of these students were in the preclinical years and they were possibly awaiting lectures on HBV.

Health care providers, who are often in frequent contact with blood and other body fluids, are well known to be at increased risk of contracting blood-borne infections such as HBV [9, 23]. Ninety and ninety-three percent of our respondents knew that health care workers could spread HBV to patients and vice versa, respectively. Though a majority of the students were aware of this risk, it is still unacceptable for some medical students to be unaware. We advocate for a hundred percent awareness prior to starting clinical rotations.

Taking the vaccine and encouraging others to take the vaccine properly require adequate knowledge on vaccine and its doses. Only 68% of our respondents correctly pointed out to the fact that the vaccine had 3 doses. This is most likely because a majority of respondents did not have the vaccine in their facilities and had not taken the vaccine.

Most respondents had appropriate knowledge on the routes of transmission of HBV. This could in a bit explain the low vaccine coverage as some respondents will turn to believe that protecting themselves against these modes of contracting the virus is less costly and sufficient.

Majority of the respondents like in other studies [24] reported having a positive attitude towards the HBV vaccine because they believe HBV is a serious disease and that the vaccine is effective in preventing the disease. This could be accounted for by the fact that attitudes are usually greatly affected by knowledge. Thus the adequate knowledge on HBV will explain the positive attitudes by respondents. Despite positive attitudes, about one-half of the respondents were worried about the adverse effects of the vaccine while another 40% were worried about the risk of acquiring infection from the vaccine. These negative attitudes could partly explain the low vaccine coverage. Emphasis should thus be made during lectures on the safety of HBV vaccine and that it has no risk of infection.

Despite providing a trove of information on HBV infection and vaccination among Cameroon medical students, our study does have limitations that are usually associated with an observational design. First, our study included only state medical schools leaving out private medical schools. Thus, this might have failed to give a true representation of the vaccine coverage among Cameroonian medical students. Also, participants' responses to negative attitudes were restricted (by predefined answers) and therefore a limited array of factors impairing vaccine receptivity in the study population was explored. More so, knowledge and attitudes were assessed using a nonstandardized questionnaire. Convenient sampling and recall bias are also shortcomings observed in our study. However the multi-institutional design of our study and large sample size increase the validity of our study.

## 5. Conclusion

Despite adequate knowledge on HBV infection, vaccination, and routes of transmission only about 1 in 6 medical students had been completely vaccinated. This low rate of vaccination may be related to unavailability of the vaccine, lack of policies on mandatory HBV vaccination, and negative attitudes towards the vaccine. Given the high prevalence of hepatitis B infection in Cameroon and the effectiveness of the HBV vaccine, eradication of this disease should be a public health priority. We recommend vaccination of those at high risk of exposure and transmission (medical students) as a first step towards eradicating this disease. This can be achieved by making the vaccine readily available and at affordable prices or free to all medical schools and hospitals. Medical school authorities should strive for 100 percent awareness among medical students and implement mandatory vaccination against HBV.

## Figures and Tables

**Table 1:** Definition of operational variables.

**Score**	**Feature**
***Knowledge on hepatitis B infection and vaccine***	
8–12 of 12 correct responses	Good knowledge on signs and symptoms
0–7 of 12 correct responses	Poor knowledge on signs and symptoms
5–7 of 7 correct responses	Good knowledge on route of transmission
0–4 of 7 correct responses	Poor knowledge on route of transmission
***Attitudes towards hepatitis B vaccine:***	
≥ 4 0f 7 Positive attitudes	Positive attitude towards HBV vaccine
≥ 3 0f 6 Negative attitudes	Negative attitude towards HBV vaccine
***HBV vaccine uptake***	
3	Complete vaccination
1–2	Incomplete vaccination
0	Not vaccinated

HBV: Hepatitis B virus.

**Table 2:** Sociodemographic characteristics of participants.

**Characteristic**	**N(total=714)**	**Percentage (%)**
**Sociodemographic**		
Females	384	53.93
Single	700	98.04
FHS Buea (clinical year)	390 (280)	54.62 (39.22)
FHS Bamenda (clinical year)	170 (90)	23.81 (12.61)
FMBS Yaoundé I (clinical year)	154 (78)	21.67 (10.92)
Basic sciences	266	37.18
Clinical year	448	62.82

N: Number of participants with the available information on the variable, FHS; Faculty of Health Sciences, FMBS; Faculty of Medicine and Biomedical Sciences.

**Table 3:** Knowledge on hepatitis B virus infection and vaccine.

**Characteristic**	**Yes (%)**	**No (%)**	**Don't know (%)**
**Knowledge on HBV and Vaccine**			
HBV can be caused by bacteria (N=706)	24 (3.40)	674 (95.47)	8 (1.13)
HBV is contagious (N=708)	552 (77.97)	138 (19.49)	18 (2.54)
HBV carrier may look healthy without showing any symptoms of the disease (N=712)	628 (88.20)	46 (6.46)	38 (5.34)
HBV can be lethal (N=712)	670 (94.10)	32 (4.49)	10 (1.40)
Patients can spread HBV to health care workers (N=710)	660 (92.96)	34 (4.79)	16 (2.25)
Health care workers can spread HBV to patients (N=704)	630 (89.49)	42 (5.97)	32 (4.55)
HBV vaccination is not for all people (N=700)	172 (24.57)	482 (68.86)	46 (6.57)
HBV vaccination does not cause hepatitis (N=700)	456 (65.14)	190 (27.14)	54 (7.71)
HBV vaccination can prevent hepatitis (N=708)	668 (94.35)	30 (4.24)	10 (1.41)
HBV vaccination does not increase the risk for complications (N=698)	498 (71.35)	106 (15.19)	94 (13.47)
HBV vaccination is contraindicated in pregnancy (N=700)	186 (16.57)	240 (34.29)	274 (39.14)
The HBV vaccine has 3 doses (N=694)	474 (68.30)	28 (4.03)	192 (27.67)
**Knowledge on route of transmission of HBV**			
Broken skin in contact with saliva contaminated with blood of HBV pos patient (N=706)	636 (90.08)	38 (5.38)	32 (4.53)
Broken skin in contact with blood of HBV pos patients (N=710)	650 (91.55)	36 (5.07)	24 (3.38)
Broken skin in contact with saliva of HBV pos patients (N=696)	484 (69.54)	138 (19.38)	74 (10.63)
Intact skin with HBV pos patient (N=702)	78 (11.11)	562 (80.06)	62 (8.83)
Intact skin with intact skin of HBV pos patient (N=704)	86 (12.22)	564 (80.11)	54 (7.67)
Needle stick injury (N=698)	584 (83.67)	64 (9.17)	50 (7.16)
Aerosol produced by a hand piece (N=688)	94 (13.66)	394 (57.27)	200 (29.07)

N: Number of participants with the available information on the variable, HBV: hepatitis B virus, pos: positive.

**Table 4:** Positive and negative attitudes towards the hepatitis B vaccine.

**Characteristic**	**Yes (%)**
**Positive attitudes**	
I am at risk because of the nature of my work (N=690)	608 (88.12)
Vaccination prevents spread of infection to patients (N=692)	584 (84.39)
Vaccination protects my family members (N=698)	634 (90.83)
Hepatitis B is a serious disease (N=698)	678 (97.13)
Hepatitis vaccine is effective in preventing the disease (N=698)	650 (93.12)
the vaccine is available at my work place (N=680)	201 (29.41)
the risk of death among vaccinated persons is reduced compared to the non vaccinated (N=690)	626 (90.72)
**Negative attitudes**	
I am worried about its adverse effects (N=690)	338 (48.99)
I am afraid of infection (N=692)	272 (39.31)
It is not effective in disease protection (N=686)	66 (9.62)
I am not at high risk of contracting HBV (N=690)	114 (16.52)
The vaccine is not available (N=688)	110 (15.99)
I have limited contact with high risk persons (N=686)	144 (22.45)

N: Number of participants with the available information on the variable.

## Data Availability

The data used to support the findings of this study are available from the corresponding author upon request.

## Abbreviations

HBV: Hepatitis B virus
HCV: Hepatitis C virus
HCP: Health care professionals
HIV: Human immunodeficiency virus
USA: United States of America
WHO: World Health Organization.

## References

[1] World Health Organisation (2015). Hepatitis B. media centre. *Fact Sheet*.

[2] Amazigo UO chime A. (1990). Hepatitis B virus infection in rural and urban populations of Eastern Nigeria, prevalence of serological markers. *East Afr Med J*.

[3] Byrne E. B. (1966). Viral hepatitis: An occupational hazard of medical personnel. Experience of the Yale-New Haven Hospital. *Journal of the American Medical Association*.

[4] Pruss-Ustün A., Rapiti E., Hutin Y. (2005). Estimation of the global burden of disease attributable to contaminated sharps injuries among health-care workers. *American Journal of Industrial Medicine*.

[5] Gambhir R., Kapoor V., Jindal G., Garg S., Setia S., Setia S. (2013). Attitudes and awareness regarding Hepatitis B and Hepatitis C amongst health-care workers of a tertiary Hospital in India. *Annals of Medical and Health Sciences Research*.

[6] CDC, Hepatitis B Virus, https://www.cdc.gov/vaccines/pubs/pinkbook/downloads/hepb.pdf

[7] Ciorlia L. A. S., Zanetta D. M. T. (2005). Hepatitis B in healthcare workers: prevalence, vaccination and relation to occupational factors. *Brazilian Journal of Infectious Diseases*.

[8] Van Damme P., Ward J. W. (2018). Hepatitis B Vaccines. *Plotkin’s Vaccines*.

[9] Moyer L. A., Mast E. E. (2001). Hepatitis B and the health care worker CDC answers frequently asked questions about how to protect health care workers. *Immun Action Coalit*.

[10] Center for Disease Control and Prevention. A comprehensive immunization strategy to eliminate transmission of hepatitis B virus infection in the United States: Recommendations of the Advisory committee on Immunization Practices (ACIP), Part 2: Immunization of adults. MMWR Recomm Rep. vol. 55, no. RR-16, 2006 17159833

[11] Shepard C. W., Simard E. P., Finelli L., Fiore A. E., Bell B. P. (2006). Hepatitis B virus infection: epidemiology and vaccination. *Epidemiologic Reviews*.

[12] Ministry of Public Health Cameroon, Plan For The Introduction of the Viral Hepatitis B Vaccine into the Routine EPI 2005–2009, pp. 1–22, 2004

[13] Von Elm E., Altman D. G., Egger M. (2007). The strengthening the reporting of observational studies in epidemiology (STROBE) statement: guidelines for reporting observational studies. *Bulletin of the World Health Organization*.

[14] Sepkowitz K. A. (1996). Occupationally acquired infections in health care workers: Part II. *Annals of Internal Medicine*.

[15] CDC. (1997). Immunization of health-care workers: recommendations of the Advisory committee on immunization practices (ACIP) and the hospital infection control practices advisory committee (HICPAC). *MMWR*.

[16] Paul N., Peterside O. (2015). Hepatitis B vaccination rate among medical students at the university of port harcourt teaching hospital (Upth). *World Journal of Vaccines*.

[17] Giri M., Panda J., Sahoo A. (2016). Hepatitis B awareness and vaccination status among first year medical students. *International Journal of Community Medicine and Public Health*.

[18] Noubiap J. J. N., Nansseu J. R. N., Kengne K. K., Tchokfe Ndoula S., Agyingi L. A. (2013). Occupational exposure to blood, hepatitis B vaccine knowledge and uptake among medical students in Cameroon. *BMC Medical Education*.

[19] Akhter M. S., Rizwan A., Wahiduzzaman M. (2016). Vaccination status and awareness of Hepatitis B among undergraduate medical students of two medical colleges in Bangladesh. *Medicine Today*.

[20] Asif M., Medicine C., College M. M. (2011). Hepatitis B vaccination coverage in medical students at a medical college of Mirpurkhas. *JPMA*.

[21] de Souza E. P., de S. Teixeira M. (2014). Hepatitis B Vaccination coverage and postvaccination serologic testing among medical students at a public university in Brazil. *Rev Inst Med Trop Sao Paulo*.

[22] Acchammachary A. A., Ubale M., Belurkar D. D., Bhave P. P., Malgaonkar A. A., Kartikeyan S. (2017). A cross-sectional study of post-vaccination anti-HBs titer and knowledge of hepatitis B infection amongst medical students in a metropolitan city. *International Journal of Research in Medical Sciences*.

[23] Ngekeng S., Chichom-Mefire A., Nde P. (2018). Hepatitis B prevalence, knowledge and occupational factors among health care workers in fako division, south west region Cameroon. *Microbiology Research Journal International*.

[24] Mariam A., Khayrat Al-Mousa J. B.

